# Perceived efficacy of case analysis as an assessment method for clinical competencies in nursing education: a mixed methods study

**DOI:** 10.1186/s12912-024-02102-9

**Published:** 2024-06-28

**Authors:** Basma Mohammed Al Yazeedi, Lina Mohamed Wali Shakman, Sheeba Elizabeth John Sunderraj, Harshita Prabhakaran, Judie Arulappan, Erna Judith Roach, Aysha Al Hashmi, Zeinab Al Azri

**Affiliations:** 1 Sultan Qaboos University, Al Khodh 66, Muscat, 123 Oman; 2 Oman College of Health Science, Norht Sharqia Branch, Ibra 66, Ibra, 124 Oman

**Keywords:** Assessment, Case-analysis, Clinical competency, Nursing education, Perception

## Abstract

**Background:**

Case analysis is a dynamic and interactive teaching and learning strategy that improves critical thinking and problem-solving skills. However, there is limited evidence about its efficacy as an assessment strategy in nursing education.

**Objectives:**

This study aimed to explore nursing students’ perceived efficacy of case analysis as an assessment method for clinical competencies in nursing education.

**Methods:**

This study used a mixed methods design. Students filled out a 13-item study-advised questionnaire, and qualitative data from the four focus groups was collected. The setting of the study was the College of Nursing at Sultan Qaboos University, Oman. Descriptive and independent t-test analysis was used for the quantitative data, and the framework analysis method was used for the qualitative data.

**Results:**

The descriptive analysis of 67 participants showed that the mean value of the perceived efficacy of case analysis as an assessment method was 3.20 (SD = 0.53), demonstrating an 80% agreement rate. Further analysis indicated that 78.5% of the students concurred with the acceptability of case analysis as an assessment method (mean = 3.14, SD = 0.58), and 80.3% assented its association with clinical competencies as reflected by knowledge and cognitive skills (m = 3.21, SD = 0.60). No significant difference in the perceived efficacy between students with lower and higher GPAs (t [61] = 0.05, *p* > 0.05) was identified Three qualitative findings were discerned: case analysis is a preferred assessment method for students when compared to MCQs, case analysis assesses students’ knowledge, and case analysis assesses students’ cognitive skills.

**Conclusions:**

This study adds a potential for the case analysis to be acceptable and relevant to the clinical competencies when used as an assessment method. Future research is needed to validate the effectiveness of case analysis exams in other nursing clinical courses and examine their effects on academic and clinical performance.

**Supplementary Information:**

The online version contains supplementary material available at 10.1186/s12912-024-02102-9.

## Introduction

Nurses play a critical role in preserving human health by upholding core competencies [1]. Clinical competence in nursing involves a constant process of acquiring knowledge, values, attitudes, and abilities to deliver safe and high-quality care [2, 3]. Nurses possessing such competencies can analyze and judge complicated problems, including those involving crucial patient care, ethical decision-making, and nurse-patient disputes, meeting the constantly altering health needs [4, 5]. To optimize the readiness of the new graduates for the challenging clinical work environment needs, nurse leaders call for integrating clinical competencies into the nursing curriculum [6, 7] In 2021, the American Association of Colleges of Nursing (AACN) released updated core competencies for professional nursing education [8]. These competencies were classified into ten fundamental essentials, including knowledge of nursing practice and person-centered care (e.g. integrate assessment skills in practice, diagnose actual or potential health problems and needs, develop a plan of care), representing clinical core competencies.

Nursing programs emphasize clinical competencies through innovative and effective teaching strategies, including case-based teaching (CBT) [9]. CBT is a dynamic teaching method that enhances the focus on learning goals and increases the chances of the instructor and students actively participating in teaching and learning [10, 11]. Additionally, it improves the students’ critical thinking and problem-solving skills and enriches their capacity for independent study, cooperation capacity, and communication skills [12–15]. It also broadens students’ perspectives and helps develop greater creativity in fusing theory and practice [16–20]. As the learning environment significantly impacts the students’ satisfaction, case analysis fosters a supportive learning atmosphere and encourages active participation in learning, ultimately improving their satisfaction [21, 22].

In addition to proper teaching strategies for clinical competencies, programs are anticipated to evaluate the students’ attainment of such competencies through effective evaluation strategies [23]. However, deploying objective assessment methods for the competencies remains challenging for most educators [24]. The standard assessment methods used in clinical nursing courses, for instance, include clinical evaluations (direct observation), skills checklists, Objective Structured Clinical Examination (OSCE), and multiple-choice questions (MCQs) written exams [25]. MCQs tend to test the recall of factual information rather than the application of knowledge and cognitive skills, potentially leading to assessment inaccuracies [26].

Given the aforementioned outcomes of CBT, the deployment of case analysis as a clinical written exam is more closely aligned with the course’s expected competencies. A mixed methods study was conducted among forty nursing students at the University of Southern Taiwan study concluded that the unfolding case studies create a safe setting where nursing students can learn and apply their knowledge to safe patient care [6]. In a case analysis, the patient’s sickness emerges in stages including the signs and symptoms of the disease, urgent care to stabilize the patient, and bedside care to enhance recovery. Thus, unfolding the case with several scenarios helps educators track students’ attained competencies [27]. However, case analysis as an assessment method is sparsely researched [28]. A literature review over the past five years yielded no studies investigating case analysis as an assessment method, necessitating new evidence. There remains uncertainty regarding its efficacy as an assessment method, particularly from the students’ perspectives [29]. In this study, we explored the undergraduate nursing students’ perceived efficacy of case analysis as an assessment method for clinical competencies. Results from this study will elucidate the position of case analysis as an assessment method in nursing education. The potential benefits are improved standardization of clinical assessment and the ability to efficiently evaluate a broad range of competencies.

## Methods

### Research design

Mixed-method research with a convergent parallel design was adopted in the study. This approach intends to converge two data types (quantitative and qualitative) at the interpretation stage to ensure an inclusive research problem analysis [30]. The quantitative aspect of the study was implemented through a cross-sectional survey. The survey captured the perceived efficacy of using case analysis as an assessment method in clinical nursing education. The qualitative part of the study was carried out through a descriptive qualitative method using focus groups to provide an in-depth understanding of the perceived strengths experienced by the students.

### Study setting

Data were collected in the College of Nursing at Sultan Qaboos University (SQU), Oman, during the Spring and Fall semesters of 2023. At the end of each clinical course, the students have a clinical written exam and a clinical practical exam, which constitute their final exam. Most clinical courses use multiple-choice questions (MCQs) in their written exam. However, the child health clinical course team initiated the case analysis as an assessment method in the clinical written exam, replacing the MCQs format.

### Participants

For this study, the investigators invited undergraduate students enrolled in the child health nursing clinical course in the Spring and Fall semesters of 2023. Currently, the only course that uses case analysis is child health. Other courses use MCQs. A total enumeration sampling technique was adopted. All the students enrolled in child health nursing clinical courses in the Spring and Fall 2023 semesters were invited to participate in the study. In the Spring, 36 students registered for the course, while 55 students were enrolled in the Fall. We included students who completed the case analysis as a final clinical written exam on the scheduled exam time. Students who did not show up for the exam during the scheduled time and students not enrolled in the course during the Spring and Fall of 2023 were excluded. Although different cases were used each semester, both had the same structure and level of complexity. Further, both cases were peer-reviewed.

### Case analysis format

The format presents open-ended questions related to a clinical case scenario. It comprises three main sections: Knowledge, Emergency Room, and Ward. The questions in the sections varied in difficulty based on Bloom’s cognitive taxonomy levels, as presented in Table 1. An answer key was generated to ensure consistency among course team members when correcting the exam. Three experts in child health nursing peer-reviewed both the case analysis exam paper and the answer key paper. The students were allocated two hours to complete the exam.

**Table 1:** Alignment of case analysis question sections with bloom’s cognitive taxonomy

Section	Description	Bloom’s Cognitive Taxonomy
1. Knowledge	Disease pathophysiology	Knowledge & understanding
2. Emergency Room questions	Findings interpretation and therapeutic management	Analysis & Evaluation
3. Ward questions	Priority nursing care plans	Application & Synthesis

### Study instruments

#### Quantitative stage

The researchers developed a study questionnaire to meet the study objectives. It included two parts. The first was about the demographic data, including age, gender, type of residence, year in the program, and cumulative grade point average (GPA). The second part comprised a 13-item questionnaire assessing the perceived efficacy of case analysis as an assessment method. The perceived efficacy was represented by the acceptability of case analysis as an assessment method (Items 1–5 and 13) and the association with clinical competencies (Items 6 to 12). Acceptability involved format organization and clarity, time adequacy, alignment with course objectives, appropriateness to students’ level, and recommendation for implementation in other clinical nursing courses. Clinical competencies-related items were relevant to knowledge (motivation to prepare well for the exam, active learning, interest in topics, collaboration while studying) and cognitive skills (critical thinking, decision-making, and problem-solving skills) (The questionnaire is attached as a supplementary document).

The questionnaire is answered on a 4-point Likert scale: 1 = strongly disagree, 2 = disagree, 3 = agree, 4 = strongly agree. Higher scores indicated better perceived efficacy and vice versa. The tool underwent content validity testing with five experts in nursing clinical education, resulting in an item-content validity index ranging from 0.7 to 1. The Cronbach alpha was 0.83 for acceptability and 0.90 for clinical competencies.

#### Qualitative stage

For the focus group interviews, the investigators created a semi-structured interview guide to obtain an in-depth understanding of the students’ perceived strengths of case analysis as an assessment method. See Table 2.

**Table 2:** Interview guide

1. 2. 3.	What are your perceived strengths of using the case analysis as an exam? What are your perceived areas for improvement in using the case analysis as an exam? Any other suggestions or recommendations to improve the case analysis as an exam?

### Data collection

#### Quantitative stage

Data was collected from the students after they gave their written informed consent. Students were invited to fill out the study questionnaire after they completed the case analysis as a clinical written exam.

#### Qualitative stage

All students in the child health course were invited to participate in focus group discussions. Students who approached the PI to participate in the focus group discussion were offered to participate in four different time slots. So, the students chose their time preferences. Four focus groups were conducted in private rooms at the College of Nursing. Two trained and bilingual interviewers attended the focus groups, one as a moderator while the other took notes on the group dynamics and non-verbal communication. The discussion duration ranged between 30 and 60 min. After each discussion, the moderator transcribed the audio recording. The transcriptions were rechecked against the audio recording for accuracy. Later, the transcriptions were translated into English by bilingual researchers fluent in Arabic and English for the analysis.

##### Rigor and trustworthiness

The rigor and trustworthiness of the qualitative method were enhanced using multiple techniques. Firstly, quantitative data, literature reviews, and focus groups were triangulated. Participants validated the summary after each discussion using member checking to ensure the moderator’s understanding was accurate. Third, the principal investigator (PI) reflected on her assumptions, experiences, expectations, and feelings weekly. In addition, the PI maintained a detailed audit trail of study details and progress. The nursing faculty conducted the study with experience in qualitative research and nursing education. This report was prepared following the Standard for Reporting Qualitative Research (SRQR) protocol [31].

### Data analysis

#### Quantitative stage

Quantitative data were entered in SPSS version 24 and analyzed using simple descriptive analysis using means, standard deviations, and percentages. After computing the means of each questionnaire item, an average of the means was calculated to identify the perceived efficacy rate. A similar technique was used to calculate the rate of acceptability and clinical competencies. The percentage was calculated based on the mean: gained score/total score* 100. In addition, the investigators carried out an independent t-test to determine the relationship between the perceived efficacy and students’ GPA.

#### Qualitative stage

The qualitative data were analyzed using the framework analysis method. In our analysis, we followed the seven interconnected stages of framework analysis: (1) transcription, (2) familiarization with the interview, (3) coding, (4) developing a working analytical framework, (5) applying the analytical framework, (6) charting data into framework matrix and (7) interpreting the data [32]. Two members of the team separately analyzed the transcriptions. Then, they discussed the coding, and discrepancies were solved with discussion.

#### Mixed method integration

In our study, the quantitative and qualitative data were analyzed separately, and integration occurred at the interpretation level by merging the data [33]. As a measure of integration between qualitative and quantitative data, findings were assessed through confirmation, expansion, and discordance. If both data sets confirmed each other’s findings, it was considered confirmation, and if they expanded each other’s insight, it was considered expansion. Discordance was determined if the findings were contradictory.

### Ethical considerations

Ethical approval was obtained from the Research and Ethics Committee of the College of Nursing, SQU (CON/NF/2023/18). Informed consent was collected, and no identifiable information was reported. For the focus group interviews, students were reassured that their grades were finalized, and their participation would not affect their grades. Also, the interviewers were instructed to maintain a non-judgmental and non-biased position during the interview. Data were saved in a locked cabinet inside a locked office room. The electronic data were saved in a password-protected computer.

## Results

The results section will present findings from the study’s quantitative and qualitative components. The integration of the two data types is described after each qualitative finding.

### Quantitative findings

We analyzed the data of 67 participants, representing a 73.6% response rate. The mean age was 21.0 years old (SD 0.73) and 36.4% were male students. See Table 3 for more details.

**Table 3:** Description of sample characteristics (*N* = 67)

Characteristics	Mean (SD) or *N* (%)
Age	21.96 (0.73)
Gender	
Male Female	16 (23.9%) 51 (76.1%)
Residence	
In campus Out campus	46 (68.7%) 21 (31.3%)
Year in the program	
Fourth Fifth	37 (55.2%) 30 (44.8%)
Cumulative GPA*	
Less or equal to 3 More than 3	33 (49.2%) 33 (49.2%)

The descriptive analysis showed that the mean value of the perceived efficacy of case analysis as an assessment method was 3.20 (SD = 0.53), demonstrating an 80% agreement rate. Further analysis indicated that 78.5% of the students concurred the acceptability of case analysis as an assessment method (mean = 3.14, SD = 0.58) and 80.3% (m = 3.21, SD = 0.60) assented the clinical competencies associated with it.

For the items representing acceptability, 81.8% of the students agreed that the case analysis was written clearly, and 80.3% reported that it was well organized. As per the questions, 81% described they were appropriate to their level, and 79.8% agreed upon their alignment with the course objectives. Moreover, the time allocated was adequate for 74.5% of the students, and 73.5% recommend using case analysis as an evaluation strategy for other clinical written examinations.

Regarding the clinical competencies, 77.3% of students agreed that the case analysis motivated them to prepare well for the exam, 81.3% reported that it encouraged them to be active in learning, and 81.0% indicated that it stimulated their interest in the topics discussed in the course. Additionally, 76.5% of the students agreed that the case analysis encouraged them to collaborate with other students when studying for the exam. Among the students, 82.5% reported that the case analysis as an assessment method enhanced their critical thinking skills, 81.0% agreed that it helped them practice decision-making skills, and 81.8% indicated that it improved their problem-solving abilities. See Table 4.

**Table 4:** Perceived effectiveness and acceptability of case analysis as clinical written exam

Item	Mean	SD	Agreement %
*The case analysis format was well-organized.*	3.21	0.71	80.3
*The case analysis format was written clearly.*	3.27	0.67	81.8
*The time allocated for answering the case analysis was adequate.*	2.98	0.91	74.5
*The questions presented in the case analysis were aligned with the course objectives.*	3.19	0.74	79.8
*The questions of the case analysis were appropriate to my level.*	3.24	0.68	81.0
*The case analysis format motivated me to prepare well for the exam.*	3.09	0.77	77.3
*The case analysis as an exam encouraged me to be active in learning.*	3.25	0.68	81.3
*The case analysis as an exam stimulated my interest in the topics discussed in the course.*	3.24	0.74	81.0
*The case analysis format encouraged me to collaborate with other students when studying for the exam.*	3.06	0.95	76.5
*The case analysis enhanced my critical thinking skills.*	3.30	0.76	82.5
*The case analysis helped me practice decision-making skills.*	3.24	0.78	81.0
*The case analysis improved my problem-solving abilities.*	3.27	0.76	81.8
*I recommend using case analysis as a strategy for clinical written examination.*	2.94	0.95	73.5

The independent t-test analysis revealed no significant difference in the perceived efficacy between students with lower and higher GPAs (t [61] = 0.05, *p* > 0.05). Further analysis showed that the means of acceptability and clinical competencies were not significantly different between the lower GPA group and higher GPA group, t [62] = 0.72, *p* > 0.05 and t [63] = -0.83, *p* > 0.05, respectively (Table 5).

**Table 5:** Independent t-test to compare the means of students with lower and higher GPA

	t	df	*P*
Perceived efficacy	0.05	61	0.958
*Acceptability*	0.72	62	0.475
*clinical competencies*	− 0.83	63	0.413

### Qualitative findings

A total of 22 had participated in four focus groups, each group had 5–6 students. The qualitative framework analysis revealed three main findings; case analysis is a preferred assessment method to students when compared to MCQs, case analysis assesses students’ knowledge, and case analysis assesses students’ cognitive skills.

#### Qualitative Finding 1: case analysis is a preferred assessment method to students when compared to MCQs

Most of the students’ statements about the case analysis as an assessment method were positive. One student stated, *“Previously, we have MCQs in clinical exams, but they look as if they are theory exams. This exam makes me deal with cases like a patient, which is good for clinical courses.”*. At the same time, many students conveyed optimism about obtaining better grades with this exam format. A student stated, *“Our grades, with case analysis format, will be better, … may be because we can write more in open-ended questions, so we can get some marks, in contrast to MCQs where we may get it right or wrong”*. On the other hand, a few students suggested adding multiple-choice questions, deleting the emergency department section, and lessening the number of care plans in the ward section to secure better grades.

Although the case analysis was generally acceptable to students, they have repeatedly expressed a need to allocate more time for this type of exam. A student stated, *“The limited time with the type of questions was a problem, …”*. When further discussion was prompted to understand this challenge, we figured that students are not used to handwriting, which has caused them to be exhausted during the exam. An example is *“writing is time-consuming and energy consuming in contrast to MCQs …”*. These statements elucidate that the students don’t necessarily mind writing but recommend more practice as one student stated, *“More experience of this type of examination is required, more examples during clinical practice are needed.”* Some even recommended adopting this format with other clinical course exams by saying *“It’s better to start this method from the first year for the new cohort and to apply it in all other courses.”*

#### Mixed Methods Inference 1: Confirmation and Expansion

The abovementioned qualitative impression supports the high acceptability rate in quantitative analysis. In fact, there is a general agreement that the case analysis format surpasses the MCQs when it comes to the proper evaluation strategies for clinical courses. Expressions in the qualitative data revealed more details, such as the limited opportunities to practice handwriting, which negatively impacted the perceived adequacy of exam time.

#### Qualitative Finding 2: case analysis assesses students’ knowledge

Students conferred that they were reading more about the disease pathophysiology, lab values, and nursing care plans, which they did not usually do with traditional means of examination. Examples of statements include *“… before we were not paying attention to the normal lab results but …in this exam, we went back and studied them which was good for our knowledge”* and *“we cared about the care plan. In previous exams, we were not bothered by these care plans”.* Regarding the burden that could be perceived with this type of preparation, the students expressed that this has helped them prepare for the theory course exam; as one student said, *“We also focus on theory lectures to prepare for this exam …. this was very helpful to prepare us for the theory final exam as well.”* However, others have highlighted the risks of limiting the exam’s content to one case analysis. The argument was that some students may have not studied the case completely or been adequately exposed to the case in the clinical setting. To solve this risk, the students themselves advocated for frequent case group discussions in the clinical setting as stated by one student: *“There could be some differences in the cases that we see during our clinical posting, for that I recommend that instructors allocate some time to gather all the students and discuss different cases.”* Also, the participants advocated for more paper-based case analysis exercises as it is helpful to prepare them for the exams and enhance their knowledge and skills.

#### Mixed Methods Inferences 2: Confirmation and Expansion

The qualitative finding supports the quantitative data relevant to items 6, 7, and 8. Students’ expressions revealed more insights, including the acquisition of deeper knowledge, practicing concept mapping, and readiness for other course-related exams. At the same time, students recommended that faculty ensure all students’ exposure to common cases in the clinical setting for fair exam preparation.

#### Qualitative Finding 3. case analysis assesses students’ cognitive skills

Several statements conveyed how the case analysis format helped the students use their critical thinking and analysis skills. One student stated, *“It, the case analysis format, enhanced our critical thinking skills as there is a case with given data and we analyze the case….”*. Therefore, the case analysis format as an exam is potentially a valid means to assess the student’s critical thinking skills. Students also conveyed that the case analysis format helped them link theory to practice and provided them with the platform to think like real nurses and be professional. Examples of statements are: *“…we connect our knowledge gained from theory with the clinical experience to get the answers…”* and *“The questions were about managing a case, which is what actual nurses are doing daily.”* Another interesting cognitive benefit to case analysis described by the students was holistic thinking. For example, one student said, *“Case analysis format helped us to see the case as a whole and not only from one perspective.”*

#### Mixed Methods Inferences 3: Confirmation

The quantitative data indicated mutual agreement among the students that the case analysis enhanced their critical thinking, decision-making, and problem-solving skills. The students’ statements from the interviews, including critical thinking, linking theory to practice, and holistic thinking, further supported these presumptions.

## Discussion

This research presents the findings from a mixed methods study that explored undergraduate nursing students’ perceived efficacy of using case analysis as an assessment method. The perceived efficacy was reflected through acceptability and association with two core competencies: knowledge and cognitive skills. The study findings showed a high rate of perceived efficacy of case analysis as an assessment method among nursing students. Additionally, three findings were extracted from the qualitative data that further confirmed the perceived efficacy: (1) case analysis is a preferred assessment method to students compared to MCQs, (2) case analysis assesses students’ knowledge, and (3) case analysis assesses students’ cognitive skills. Moreover, the qualitative findings revealed details that expanded the understanding of the perceived efficacy among nursing students.

Previous literature reported students’ preference for case analysis as a teaching method. A randomized controlled study investigated student’s satisfaction levels with case-based teaching, in addition to comparing certain outcomes between a traditional teaching group and a case-based teaching group. They reported that most students favored the use of case-based teaching, whom at the same time had significantly better OSCE scores compared to the other group [34]. As noted, this favorable teaching method ultimately resulted in better learning outcomes and academic performance. Although it may be challenging since no answer options are provided, students appreciate the use of case analysis format in their exams because it aligns better with the course objectives and expected clinical competencies. The reason behind students’ preference for case analysis is that it allows them to interact with the teaching content and visualize the problem, leading to a better understanding. When case analysis is used as an assessment method, students can connect the case scenario presented in the exam to their clinical training, making it more relevant.

In this study, students recognized the incorporation of nursing knowledge in the case analysis exam. They also acknowledged improved knowledge and learning abilities similar to those observed in case-based teaching. Boney et al. (2015) reported that students perceived increased learning gains and a better ability to identify links between different concepts and other aspects of life through case-based teaching [35]. Additionally, case analysis as an exam promotes students’ in-depth acquirement of knowledge through the type of preparation it entails. Literature suggested that case-based teaching promotes self-directed learning with high autonomous learning ability [34, 36]. Thus, better achievement in the case analysis exam could be linked with a higher level of knowledge, making it a suitable assessment method for knowledge integration in nursing care.

The findings of this study suggest that case analysis can be a useful tool for evaluating students’ cognitive skills, such as critical thinking, decision-making, and problem-solving. A randomized controlled study implied better problem-solving abilities among the students in the case-based learning group compared to those in the traditional teaching methods group [12]. Moreover, students in our study conveyed that case analysis as an exam was an opportunity for them to think like real nurses. Similar to our findings, a qualitative study on undergraduate nutrition students found that case-based learning helped students develop professional competencies for their future practice, in addition to higher-level cognitive skills [37]. Therefore, testing students through case analysis allows educators to assess the student’s readiness for entry-level professional competencies, including the thinking process. Also, to evaluate students’ high-level cognitive skills according to Bloom’s taxonomy (analysis, synthesis, and evaluation), which educators often find challenging.

Case analysis as an assessment method for clinical courses is partially integrated in case presentation or OSCE evaluation methods. However, the written format is considered to be more beneficial for both assessment and learning processes. A qualitative study was conducted to examine the impact of paper-based case learning versus video-based case learning on clinical decision-making skills among midwifery students. The study revealed that students paid more attention and were able to focus better on the details when the case was presented in a paper format [38]. Concurrently, the students in our study recommended more paper-based exercises, which they believed would improve their academic performance.

This study has possible limitations. The sample size was small due to the limited experience of case analysis as a clinical written exam in the program. Future studies with larger sample sizes and diverse nursing courses are needed for better generalizability.

### Implications

Little evidence relates to the efficacy of case analysis as an evaluation method, suggesting the novelty of this study. Despite the scarcity of case-based assessment studies, a reader can speculate from this study’s findings that there is a potential efficacy of case analysis as an assessment method in nursing education. Future research is warranted to validate the effectiveness of case-analysis assessment methods and investigate the effects of case-analysis exams on academic and clinical performance.

## Conclusions

Overall, our findings are in accordance with the evidence suggesting students’ perceived efficacy of case analysis as a teaching method. This study adds a potential for the case analysis to be acceptable and relevant to the clinical competencies when used as an assessment method. Future research is needed to validate the effectiveness of case analysis exams in other nursing clinical courses and examine their effects on academic and clinical performance.

### Electronic supplementary material

Below is the link to the electronic supplementary material.

**Supplementary Material 1:** The questionnaire used in this study is attached as a supplementary document.

Supplementary Material 2

## Data Availability

The datasets used and/or analyzed during the current study are available fromthe Principal Investigator (BAY) upon reasonable request.
