# Supplementary material for: Perceived efficacy of case analysis as an assessment method for clinical competencies in nursing education: a mixed methods study
Source: BMC Nurs. 2024 Jun 28;23:441. doi: 10.1186/s12912-024-02102-9 (PMC11212368; doi:10.1186/s12912-024-02102-9)
Supplement: Supplementary file 1 — Supplementary Material 1: The questionnaire used in this study is attached as a supplementary document. [file 12912_2024_2102_MOESM1_ESM.pdf]

Sultan Qaboos University

College of Nursing

"Students' Perception of Using Case Analysis as a Clinical Written Exam"

**Section A. General Information.**<br>*Please circle the option relevant to you.	
**1. Age:**	……years
**2. Gender:**	A. Male<br>B. Female
**3. Residence:**	A. In campus<br>B. Off-campus
**4. Year in the program:**	A. 3rd year<br>B. 4th year<br>C. 5th year<br>D. Other…..
**5. Cumulative GPA:**	A. Less than 2<br>B. 2- 2.5<br>C. 2.6 – 3<br>D. 3.1- 3.5<br>E. Above 3.5

**Section B. Students' Perception of Using Case Analysis as a Clinical Written Exam Questionnaire.**<br>*Students' instruction: Please rate each of the following items from your perspective. There are four options to choose from; strongly agree (4), agree (3), disagree (2), and strongly disagree (1).*					
**No**	**Items**	*Strongly Agree (4)*	*Agree (3)*	*Disagree (2)*	*Strongly Disagree (1)*
**1.**	The case analysis format was well-organized.				
**2.**	The case analysis format was written clearly.				
**3.**	The time allocated for answering the case analysis was adequate.				
**4.**	The questions presented in the case analysis were aligned with the course objectives.				
**5.**	The questions of the case analysis were appropriate to my level.				
**6.**	The case analysis format motivated me to prepare well for the exam.				
**7.**	The case analysis as an exam encouraged me to be active in learning.				
**8.**	The case analysis as an exam stimulated my interest in the topics discussed in the course.				
**9.**	The case analysis format encouraged me to collaborate with other students when studying for the exam.				
**10.**	The case analysis enhanced my critical thinking skills.				
**11.**	The case analysis helped me practice decision-making skills.				
**12.**	The case analysis improved my problem-solving abilities.				
**13.**	I recommend using case analysis as a strategy for clinical written examination.				

Your general feedback.

…………………………………………………………………………………………………
…………………………………………………………………………………………………
…………………………………………………………………………………………………
…………………………………………………………………………………………………
…………………………………………………………………………………………………
…………………………………………………………………………………………………
…………………………………………………………………………………………………
…………………………………………………………………………………………………
…………………………………………………………………………………………………
…………………………………………………………………………………………………
…………………………………………………………………………………………………
…………………………………………………………………………………………………
…………………………………………………………………………………………………
…………………………………………………………………………………………………
…………………………………………………………………………………………………
…………………………………………………………………………………………………
