# Supplementary material for: Perceived efficacy of case analysis as an assessment method for clinical competencies in nursing education: a mixed methods study
Source: BMC Nurs. 2024 Jun 28;23:441. doi: 10.1186/s12912-024-02102-9 (PMC11212368; doi:10.1186/s12912-024-02102-9)
Supplement: Supplementary file 2 — Supplementary Material 2 [file 12912_2024_2102_MOESM2_ESM.pdf]

PI. Dr. Basma Al Yazeedi
Action Research

Consent to Participate in a Research Study

Sultan Qaboos University, College of Nursing
Title of Study: Perception of Using Case Analysis Format as a Clinical Written Exam
Principal Investigator: Dr. Basma Alyazeedi
Principal Investigator Department: Maternal and Child Health
Principal Investigator Phone number: 24145415
Principal Investigator Email Address: basma84@squ.edu.om

What are some general things you should know about research studies?

You are being asked to take part in a research study. To join the study is voluntary. You may refuse to join, or you may withdraw your consent to be in the study without penalty. You should ask the researcher named above any questions you have about this study at any time.

What is the purpose of this study?

The purpose of this study is to explore the perceptions around using case analysis as a clinical written exam compared to the traditional multiple choice questions format. You are selected as a possible participant in this study because you are enrolled in the NURS3021 Child Health Nursing Clinical Course at Sultan Qaboos University, Sultanate of Oman at the time of the study.

What will happen if you take part in the study?

Participation in the study will involve filling a questionnaire and undergoing an interview. The interview will be in a group.

- **Questionnaire**: A 13-item questionnaire will be introduced to assess the participants' perception on the case analysis exam format acceptability and advantages as a clinical written exam.

- **Interview:** Few open-ended questions to reflect participants' opinion and suggestions to improve the applicability of the exam format will be asked.

What are the possible benefits from being in this study?

Research is designed to benefit society by gaining new knowledge. Your critical thinking, problem solving skills, as well as your motivation for self-directed learning may or may not improve. The information obtained from this study will help shape future well-designed clinical written exams.

What are the possible risks or discomforts involved from being in this study?

There is no risk involved in this research study.

How will information about you be protected?

Data collected in this study will be saved in a locked cabinet inside a locked room and data entered in computers will be password protected. The research team will only have the access to the data collected in this study. Data will be analyzed, and results will be published in nursing education journals. No identifying information such as names or birth dates will be asked or published.

Participant's Agreement:
I have read the information provided above and asked questions that I have at this time, if any. I voluntarily agree to participate in this research study.

____ ____________

Signature of Research Participant Date

____ ____________

Signature of Research Team Member Obtaining Consent Date
